# Improving Semantic Segmentation of Urban Scenes for Self-Driving Cars with Synthetic Images

**DOI:** 10.3390/s22062252

**Published:** 2022-03-14

**Authors:** Maksims Ivanovs, Kaspars Ozols, Artis Dobrajs, Roberts Kadikis

**Affiliations:** 1 Institute of Electronics and Computer Science, 14 Dzerbenes St., LV-1006 Riga, Latvia; kaspars.ozols@edi.lv (K.O.); roberts.kadikis@edi.lv (R.K.); 2 SIA Mondot, 80A Balasta Dambis, LV-1048 Riga, Latvia; artis.dobrajs@mondot.lv

**Keywords:** self-driving cars, semantic segmentation, deep learning, synthetic data, CARLA, Cityscapes

## Abstract

Semantic segmentation of an incoming visual stream from cameras is an essential part of the perception system of self-driving cars. State-of-the-art results in semantic segmentation have been achieved with deep neural networks (DNNs), yet training them requires large datasets, which are difficult and costly to acquire and time-consuming to label. A viable alternative to training DNNs solely on real-world datasets is to augment them with synthetic images, which can be easily modified and generated in large numbers. In the present study, we aim at improving the accuracy of semantic segmentation of urban scenes by augmenting the Cityscapes real-world dataset with synthetic images generated with the open-source driving simulator CARLA (Car Learning to Act). Augmentation with synthetic images with a low degree of photorealism from the MICC-SRI (Media Integration and Communication Center–Semantic Road Inpainting) dataset does not result in the improvement of the accuracy of semantic segmentation, yet both MobileNetV2 and Xception DNNs used in the present study demonstrate a better accuracy after training on the custom-made CCM (Cityscapes-CARLA Mixed) dataset, which contains both real-world Cityscapes images and high-resolution synthetic images generated with CARLA, than after training only on the real-world Cityscapes images. However, the accuracy of semantic segmentation does not improve proportionally to the amount of the synthetic data used for augmentation, which indicates that augmentation with a larger amount of synthetic data is not always better.

## 1. Introduction

Self-driving cars, also known as robotic cars [1], autonomous vehicles [2,3], and driverless vehicles [4], are currently one of the most promising emerging technologies and a lively area of research. Research labs, universities, and companies have been actively working on self-driving cars since the mid-1980s [5]; in the last decade, research on self-driving cars and development of their prototypes have been gaining pace with an increasing focus on applying technologies related to data gathering and processing [6]. However, the task of developing self-driving cars with the highest level of autonomy, i.e., autonomous to such an extent that no human intervention is required in any circumstances [7], still remains an unsolved challenge.

The architecture of the autonomy system of self-driving cars is typically organised into two main parts: the perception system and the decision-making system [8]. Some of the tasks that the perception system deals with are object recognition, object localisation, and semantic segmentation [9]. In particular, semantic segmentation, also known as scene parsing [10], aims to classify every pixel of the image [11]; in other words, it is an image classification task at the pixel level [12]. Similar to image classification [13] and object detection [14], state-of-the-art semantic segmentation results have been achieved with deep neural networks (DNNs) [15,16,17], which, therefore, are particularly pertinent to the design of the navigation system of self-driving cars [18]. However, despite their impressive performance on many perceptual tasks, DNNs also have shortcomings: their inner workings are not transparent, as they operate as black boxes [19,20], they require a lot of computational power for running inference [9,21], and training them takes a lot of time, computing resources, especially GPU (graphics processing unit) power, and data [22]. Among these considerations, the need for large training datasets is arguably especially topical for deep learning, as data acquisition takes a lot of money and person-hours as well as may be hampered by legal obstacles and privacy concerns. These problems are even more pertinent to the acquisition of the datasets of street views for semantic segmentation that are needed for training DNN models for the perception systems of self-driving cars, as it is expensive and time-consuming to acquire images in the urban setting, and sharing the acquired data publicly can be legally challenging. Furthermore, pixel-wise labelling takes a lot of time and effort: thus, for the creation of the Cambridge-driving Labeled Video Database (CamVid dataset) [23], labelling was reported to take around 1 h per image; in the case of the Cityscapes dataset [24], fine pixel-level annotation and quality control of a single image required on average more than 1.5 h. Therefore, it is not surprising that publicly available urban street view datasets with semantic labelling are comparatively small in size: CamVid [23] consists of 700 annotated images obtained from a video sequence of 10 min; the pixel-wise labelled subset of DUS (Daimler Urban Segmentation Dataset) [25] is 500 images large; in Cityscapes [24], there are 5000 fine-labelled and 20,000 coarse-labelled images. The scarcity of semantically labelled data for self-driving cars caused by the “curse of dataset annotation" [26] and the major problem for training DNN models for semantic segmentation in general may arguably hinder the development of self-driving cars with a high degree of autonomy.

One of the approaches to dealing with the problem of the scarcity of real-world images is to resort to synthetic data, i.e., artificially generated data that are at least to some extent similar to real-world data. The use of synthetic data not only obviates the need to acquire real-world data, but also offers further advantages. Thus, a pipeline for the generation of synthetic images can be modified to produce more diverse data, e.g., by changing the weather conditions or increasing the number of objects of interest such as cars, pedestrians, or traffic signs; therefore, it is possible to produce diverse synthetic data on a large scale. Furthermore, such pipelines usually make it unnecessary to annotate generated images manually, as the contours of the objects and the background can be obtained automatically and with a high degree of precision.

Because of these advantages, synthetic images have been successfully used for various tasks related to self-driving cars including semantic segmentation. Thus, Ros et al. [27] used synthetic images from their SYNTHIA (SYNTHetic collection of Imagery and Annotations) dataset, which represents a virtual New York City modelled by the authors with the Unity platform, to augment real-world datasets and improve semantic segmentation; Richter et al. [28] extracted synthetic images and the data for generating semantic segmentation masks from the video game Grand Theft Auto V and used the acquired synthetic dataset to improve the accuracy of semantic segmentation; Hahner et al. [29] used a custom-made dataset of synthetic images of foggy street scenes to improve the quality of semantic scene understanding under foggy road conditions. However, there are still several challenges in the use of synthetic data for self-driving cars. First, even high-quality synthetic images are not entirely photorealistic and therefore are less valuable for training than real-world images; therefore, it is often more reasonable to augment real-world data with synthetic data rather than to train DNN models on synthetic data alone [30]. Second, generation of synthetic data can require quite a lot of effort—at least when designing the initial setup: thus, developing a pipeline for the generation of synthetic images can be challenging in terms of time and effort involved, while the acquisition of synthetic images from video games may not be an easy task either, as the internal workings and assets of the games are usually inaccessible [28].

A promising alternative path for the generation of synthetic images is to use open-source sandbox driving simulators such as TORCS (The Open Racing Car Simulator) [31] or CARLA (Car Learning to Act) [32]. While such simulators are said to lack the extensive content of the worlds from the top-level video games [28], their open-source nature makes it easier to access and modify them as well as imposes little (if any) legal constraints on the use of the eventually generated data. Recently, Berlincioni et al. [33] made use of the data produced with a driving simulator: the authors generated their MICC-SRI (Media Integration and Communication Center–Semantic Road Inpainting) dataset with CARLA to tackle the problem of image inpainting [34,35], i.e., predicting missing or damaged parts of an image by inferring them from the context. However, it is still an open question whether the quality of the synthetic images generated with open-source driving simulators is sufficient to improve the accuracy of DNNs on semantic segmentation, a task with particularly high requirements for the quality of the training data.

The present study is concerned with improving the quality semantic segmentation of urban scenes by means of augmenting a dataset of real-world images with synthetic images. In particular, the goal of the study is to investigate whether it is possible to achieve improvement in the accuracy of semantic segmentation by using synthetic data generated with the open-source driving simulator CARLA [32], which can be done in a relatively simple and fast manner. While the need for a lot of computation time for training the DNN models still remains a challenge, we dramatically decrease the time (i.e., the person-hours) needed for the generation of the synthetic data. We perform experiments with DNNs from the DeepLab model library [36]; the source dataset of the real-world images is Cityscapes [24], currently the default benchmark dataset for semantic segmentation [37,38,39,40]. In the first series of experiments, we augment Cityscapes with the already available CARLA-generated images from the MICC-SRI dataset [33] and train MobileNetV2 [41] and Xception [42] DNNs on the augmented dataset. To provide a baseline for the assessment of the performance of the models trained on the augmented dataset, we also train MobileNetV2 and Xception models on the original Cityscapes real-world images alone. For the second series of experiments, we create our own CCM (Cityscapes-CARLA Mixed) dataset, the real-world part of which consists of Cityscapes images, whereas the synthetic part of it is generated with CARLA. As we use a more recent (v0.9.12 vs. v0.8.2) version of CARLA than in [33], we generate images with higher resolution, improved photorealism, and more diverse assets—both static (e.g., buildings, traffic signs) and dynamic (e.g., cars and pedestrians)—than in the MICC-SRI dataset. We train both MobileNetV2 and Xception on the CCM dataset as well as (again, to provide a baseline for the comparison) on the original Cityscapes real-world images alone. Furthermore, to investigate the relation between the amount of synthetic data used for augmentation and the accuracy of the semantic segmentation, we train MobileNetV2 and Xception on several splits of the CCM dataset, each of which contains all real-world Cityscapes images and a varying part (100, 50, and 25 percent) of the available synthetic images. As a result of experiments on the augmented CCM datasets, we obtain DNN models with MobileNetV2 and Xception architectures that perform semantic segmentation better than their counterparts trained solely on the real-world Cityscapes images; these models can be used for the development of the prototype of a self-driving car. Furthermore, we demonstrate that it is possible to improve the quality of semantic segmentation of street views by rather simple means, as running a simulation of driving along the streets and saving the frames of the video stream in CARLA is obviously much easier than virtually recreating parts of New York [27] or San Francisco [43], or acquiring the frames by intercepting communication between the software program of a video game and its hardware [28,37,44]. Finally, our findings also bear relevance to the general methodology of the use of synthetic data for augmentation, as they demonstrate that a larger amount of synthetic data does not necessarily result in better semantic segmentation.

The rest of the paper is organised as follows. Section 2 describes materials and methods of the present study; Section 3 is concerned with its main results and their discussion; finally, Section 4 presents the conclusions.

## 2. Materials and Methods

### 2.1. Datasets

**Figure 1:**
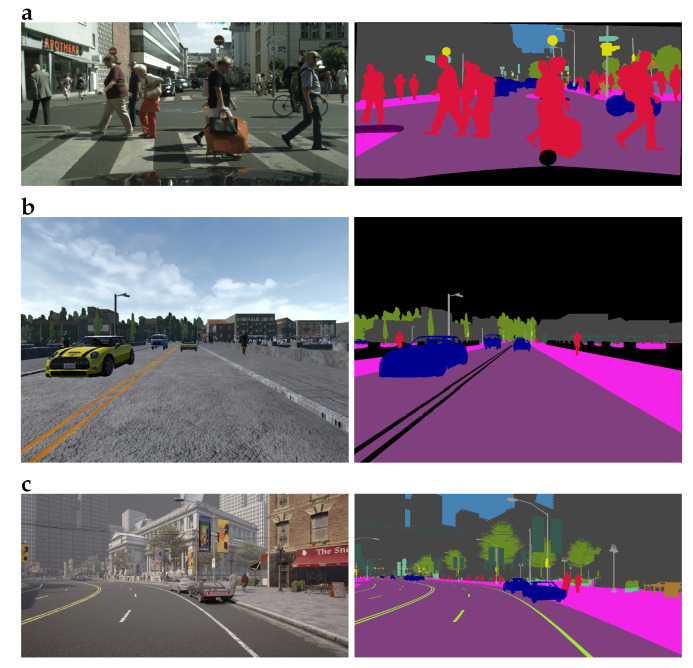
Sample images and their segmentation masks from the datasets: (**a**) Cityscapes dataset; (**b**) MICC-SRI (Media Integration and Communication Center–Semantic Road Inpainting) dataset; (**c**) CCM (Cityscapes-CARLA Mixed) dataset. Note that (**a**,**c**) are not to scale with respect to (**b**), as the actual resolution of (**a**) and (**a**) is 1024 × 2048 pixels vs. 600 × 800 pixels for (**b**).

Three datasets were used in the present study: the Cityscapes dataset [24] of real-world images, the MICC-SRI dataset [33] composed of synthetic images generated with the CARLA simulator, and our custom-made CCM dataset. These three datasets are described in detail in the following; sample images from each dataset and their segmentation masks are shown in Figure 1.

Cityscapes [24] is one of the most well-known datasets of urban landscapes for self-driving cars. It comprises a diverse set of images with the resolution of 1024 by 2048 pixels taken in the streets of 50 different European (predominantly German) cities while driving a specially equipped car. Coarse semantic segmentation annotations are available for 20,000 of these images, and fine (pixel-level) annotations are available for 5000 images. In the present study, we used only those Cityscapes images for which fine annotations are available. Furthermore, we had to change the split of the original dataset into training, validation, and test sets, as due to relabelling of the segmentation masks (see Section 2.2) to ensure compatibility of Cityscapes images with the synthetic images generated with CARLA, it was not possible to benchmark our models on the Cityscapes test set, which is withheld from the public access to ensure impartial benchmarking. Our custom split of the original Cityscapes dataset was as follows: out of 3475 Cityscapes fine-annotated images available in public access, we used 2685 images for training, 290 images for validation, and 500 images for the final testing of the DNN models we trained. As in the original Cityscapes data split reported by its authors [24], images from a particular location would appear only in one of the sets, i.e., in training, or validation, or test set, but not in two or all three of them. The purpose of that was to ensure a strict separation between the data in each of the three subsets, which was necessary to prevent a methodologically unsound situation when a DNN model would be tested on the data too similar to the data it was trained on. More specifically, we used images taken in Frankfurt, Lindau, and Münster locations for the test dataset, images taken in Bochum, Krefeld, and Ulm locations for the validation dataset, and the rest of the images for the training dataset.

The MICC-SRI dataset [33] consists of 11,913 synthetic RGB frames of urban driving footage with the resolution of 600 by 800 pixels generated with the CARLA simulator [32]; for all RGB frames, semantic segmentation annotations are provided. The dataset was originally created for semantic road inpainting tasks, and RGB images in it are not photorealistic (cf. Figure 1b). As Berlincioni et al. [33] report, the frames for the MICC-SRI dataset were collected by running a different simulation for 1000 frames from each spawning point of the two available maps in the most recent CARLA version at the time of conducting the study, v0.8.2. Simulations were run at 3 FPS; to ensure diversity of the generated data, the simulations were subsampled to take an image every 3 s. Further processing of the generated data reported by the authors [33] included removing occasional misalignment between the RGB frames and segmentation masks. RGB frames and corresponding semantic segmentation annotations in the MICC-SRI dataset are available in two versions, the one with the static objects only, and the one with both static and dynamic (cars and pedestrians) objects. For the purpose of our study, we used only the RGB images and segmentation masks with both static and dynamic objects in them.

Our custom-made CCM dataset consists of 2685 Cityscapes images as well as 46,935 synthetic images that we generated with the CARLA simulator. The resolution of the synthetic images is 1024 by 2048 pixels (i.e., matching Cityscapes’s image resolution); the images were collected by running simulations of several maps available in the latest stable release of CARLA (v0.9.12), namely, Town 1, Town 2, Town 3, Town 4, and Town 10. The simulations were run at 1 FPS; an RGB image and its respective segmentation mask were saved every second. To make the settings more diverse, simulation in Town 1 was run with the weather set to “Clear Noon”, whereas simulation in Town 10 was run with the setting “Cloudy Noon”; on the rest of the maps, the simulations were run with default settings. To acquire images with a large number of dynamic objects in them, the simulations were run with the number of vehicles spawned in them set to 100, and the number of pedestrians spawned in them set to 200. All in all, the number of acquired images in different locations was as follows: in Town 1, 7866 images; in Town 2, 3838 images; in Town 3, 11,124 images; in Town 4, 11,484 images; in Town 10, 13,049 images. The overall time of running simulations for image acquisition was approximately 96 h on a desktop PC with OS Windows 10, Intel i5-6400 CPU, and NVIDIA 1060 GPU.

### 2.2. Data Preprocessing

Data preprocessing consisted of relabelling the semantic segmentation masks, resizing images, and augmenting real-world Cityscapes data with synthetic data. All these procedures are described in the following.

A relabelling of the semantic segmentation masks was done to ensure compatibility between the annotation labels of Cityscapes images and CARLA images in the MICC-SRI dataset as well as between annotation labels of the Cityscapes images and CARLA images in the CCM dataset. It was necessary to use different label mappings for the former and the latter case, because CARLA images in the MICC-SRI dataset were generated with CARLA v0.8.2, which has fewer labels than the more recent CARLA v0.9.12, which was used for generating synthetic data for the CCM dataset. Mapping between labels in Cityscapes and MICC-SRI was the same as in experiments in [33]; as it is not reported in detail in the original publication [33], we provide it in Table 1.

The mapping between Cityscapes labels and CARLA v0.9.12 labels that was designed in order to create the CCM dataset is reported in Table 2.

Image resizing was required for the experiments on the Cityscapes and MICC-SRI datasets, as the images in these datasets were of different size (1024 × 2048 pixels vs. 600 × 800 pixels, respectively), whereas the input to a DNN should typically be of a uniform size. One possible solution was to upscale MICC-SRI images; however, as they have different height-to-width ratios than the Cityscapes dataset (0.75:1 vs. 0.5:1), that would result in a notable distortion of the images and would likely lead to a decrease in the accuracy of the semantic segmentation. Therefore, we used the opposite approach and split each Cityscapes image into 9 smaller images with the size of 600 × 800 pixel, i.e., the same as the images in the MICC-SRI dataset. There was no need for such resizing for the experiments involving the CCM and Cityscapes datasets, because synthetic images in the former dataset were generated with the same size as images in the latter dataset.

Finally, to investigate how the amount of the synthetic data used for augmentation affected the accuracy of the semantic segmentation, we created three splits of the CCM dataset, each of which included all Cityscapes real-world images designated for training and, respectively, 100, 50, and 25 percent of synthetic images that we generated with the CARLA simulator. Synthetic images for splits were selected randomly; to avoid unnecessary verbosity, we henceforth refer to these splits as CCM-100, CCM-50, and CCM-25.

### 2.3. DNNs for Semantic Segmentation and Training Procedure

We conducted semantic segmentation experiments with two DNN models from the DeepLab library [36], MobileNetV2 [41] and Xception [42], both pretrained on the PASCAL VOC 2012 dataset [45]. The DeepLab library is a well-known state-of-the-art library for semantic segmentation; we chose these two particular models from it, as MobileNetV2 is a compact and fast convolutional neural network (CNN), whereas Xception is a larger CNN offering better segmentation accuracy at the cost of longer training time and larger requirements for GPU memory size. The models were trained on high-performance computing (HPC) Dell EMC PowerEdge C4140 servers equipped with Intel Xeon Gold 6130 CPU and NVIDIA V100 GPU with 16 GB VRAM memory. We used the default settings of the models for training: for MobileNetV2 models, the output stride was set to 8, and the training crop size was 769 by 769 pixels; for Xception models, the atrous rates were set to 6, 12, and 18, the output stride was 16, the decoder output stride 4, and the training crop size was 769 by 769 pixels. The learning rate was set to 0.0001 for all models, and the learning was optimized with the SGD with momentum optimizer, with the momentum value set to 0.9.

We used only real-world images for validating and testing the models, i.e., in all experiments, the validation dataset was the Cityscapes validation set, and the test dataset was the Cityscapes test set. The batch size for training was the maximum batch size possible with the size of the GPU memory at our disposal, namely, 4 images for MobileNetV2 models and 2 images for Xception models. For experiments on the MICC-SRI dataset and the Cityscapes dataset, the MobileNetV2 model was trained for 1200 epochs, and the Xception model was trained for 300 epochs on each of these datasets. For experiments on the CCM dataset and the Cityscapes dataset, the MobileNetV2 and Xception models were trained for 200 epochs on the Cityscapes dataset and on CCM-100, CCM-50, and CCM-25 dataset splits. All in all, training two models for experiments on the MICC-SRI dataset and the Cityscapes dataset took approximately 1480 h of computing, whereas training eight models for experiments on the Cityscapes dataset and the splits of the CCM dataset took around 1415 h of computing.

## 3. Results and Discussion

We report our main results with the standard metric for semantic segmentation, intersection over union (IoU), which is also known as the Jaccard Index, as well as the mean intersection over union (mIoU). The same as other authors, e.g., Cordts et al. [24], we report and include in the mIoU calculations only semantically meaningful classes, excluding such classes as “Other” or “None”. As we had to change the labelling scheme from the one originally used in the Cityscapes dataset, we cannot directly compare the performance of the DNN models trained on the augmented datasets with the state-of-the-art results on the original Cityscapes dataset reported in the literature [36]. Therefore, we compared the performance of the models trained on the augmented datasets with that of the DNN models with the same architecture that we trained on the original Cityscapes dataset with the accordingly modified map of labels.

### 3.1. Results on Cityscapes and MICC-SRI Datasets

We summarise the main results of training the MobileNetv2 and Xception DNN models on the Cityscapes and MICC-SRI datasets in Table 3 and Table 4. As can be seen, augmentation of the Cityscapes dataset with MICC-SRI images did not improve the accuracy of the semantic segmentation: just the opposite, both MobileNetV2 and Xception models perform better when trained only on real-world images that on the dataset augmented with synthetic images, with an mIoU 75.43% vs. 75.11% for the MobileNetV2 model and an mIoU of 79.34% and 78.81% for the Xception model. The MobileNetV2 model trained on the real-world images only performs better than its counterpart trained on the augmented dataset across all segmentation classes, whereas in the case of the Xception models, the only class on which the model trained on the augmented data does better than its counterpart trained on the real-world images is “Sidewalk”. The likely explanation for the worse performance of the models trained on the augmented dataset is the low photorealism of images in the MICC-SRI dataset: while the quality images were sufficient for the semantic road inpainting task in [33], it turned out to not be good enough for the task of semantic segmentation. It should also be observed that the Xception models trained on the Cityscapes and MICC-SRI datasets demonstrate better performance than the MobileNetV2 models trained on the same datasets with an mIoU 79.34% vs. 75.43% on the Cityscapes dataset and mIoU 78.81% vs. 75.11% on the MICC-SRI dataset. Such a difference in performance is likely due to the larger size of the Xception model.

### 3.2. Results on Cityscapes and CCM Datasets

The results from training the MobileNetV2 and Xception models on Cityscapes and the three splits of the CCM dataset, CCM-100, CCM-50, and CCM-25 datasets are reported in Table 5 and Table 6, respectively. As can be seen, for both DNN architectures, augmentation with synthetic data improves the accuracy of the semantic segmentation. Thus, for MobileNetV2, the model trained on CCM-100 achieves an mIoU of 55.49%, the model trained on CCM-50 achieves an mIoU of 56.05%, and the model trained on CCM-25 achieves an mIoU of 52.67%, whereas the model trained on Cityscapes images only achieves an mIoU of 44.68%. The same applies to Xception models: namely, the model trained on CCM-100 achieves an mIoU of 63.14%, the model trained on CCM-50 achieves an mIoU of 63.87%, and the model trained on CCM-25 achieves an mIoU of 64.46%, whereas the model trained on Cityscapes images only achieves an mIoU of 57.25%. It is remarkable that the best-performing MobileNetV2 and Xception models are not the ones trained on the splits of the CCM dataset with the largest amount on synthetic data, as the best-performing MobileNetV2 model is the one trained on CCM-50, while the best-performing Xception model is the one trained on the split with the smallest amount of synthetic data, that is, CCM-25. That indicates that a larger amount of synthetic data used for augmentation does not necessarily result in better performance than augmentation with a smaller amount of synthetic data.

**Figure 2:**
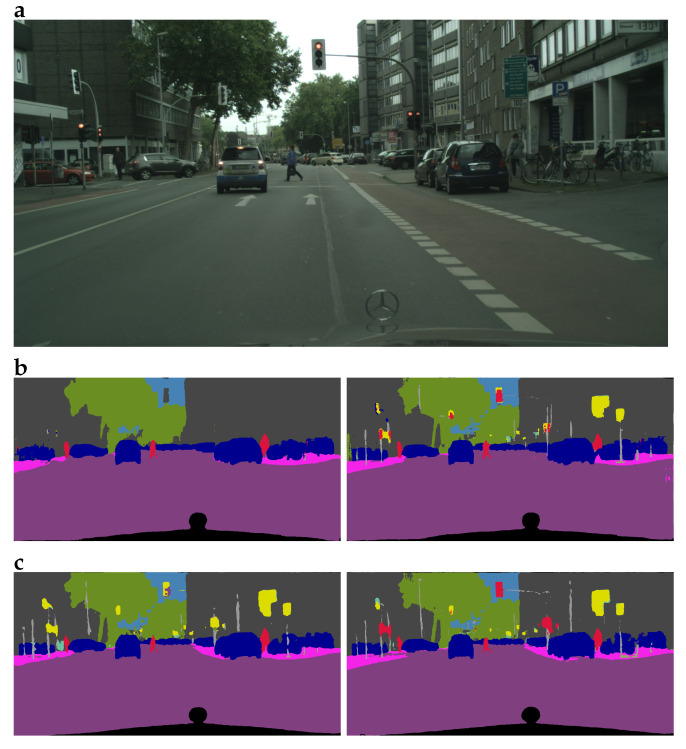
Semantic segmentation of a sample image with different models: (**a**) the original image; (**b**) segmentation masks produced with MobileNetV2 models: trained on Cityscapes images only (**left**) and on CCM-50 split (**right**); (**c**) segmentation masks produced with Xception models: trained on Cityscapes images only (**left**) and on CCM-25 split (**right**). Note the differences in the ability of the models to classify traffic lights and road signs.

Another finding worth noting is that models trained on different splits of the CCM dataset show best results (i.e., in comparison to other models) on different classes: thus, the model trained on CCM-100 performs better than other models on the classes “Traffic Sign” and “Traffic Lights”; the model trained on CCM-50—on the classes “Building”, “Fence”, “Pole”, “Road”, “Sidewalk”, “Vegetation”, “Wall”, and “Water and Terrain”; finally, the model trained on CCM-25 outperforms models trained on other splits on the classes “Pedestrian and Rider”, “Vehicles”, and “Sky”. These differences are exemplified in Figure 2. In particular, the MobileNetV2 model trained only on the real-world Cityscapes images cannot classify road signs and traffic lights; as for the Xception model trained only on the real-world Cityscapes images, while it can classify road signs, it is not capable of distinguishing between traffic lights and road signs, mistakenly classifying the objects from the former class as belonging to the latter class. Contrary to that, MobileNetV2 and Xception models trained, respectively, on CCM-50 and CCM-25 splits, i.e., the best-performing models for their respective architectures, are quite capable of classifying road signs and traffic lights and distinguishing between these two types of objects. These observations correspond to the results in Table 5 and Table 6 regarding the ability of the models to classify the classes “Traffic Lights” and “Road Signs”.

## 4. Conclusions

The goal of the study was to investigate whether it is possible to improve the quality of semantic segmentation by augmenting real-world images of urban scenes with synthetic images generated with the open-source driving simulator CARLA. We used the Cityscapes dataset as our source dataset of the real-world images and performed experiments with two DNN architectures from the Deeplab library, MobileNetV2 and Xception. We conducted two series of experiments, which altogether took approximately 2895 h of computing on a rather powerful server equipped with an NVIDIA V100 GPU. In the first series of experiments, we augmented the Cityscapes dataset with synthetic images from the MICC-SRI dataset; however, because of the low degree of photorealism of the synthetic images, the augmentation led to the deterioration rather than to a better accuracy of the semantic segmentation. In the second series of experiments, we augmented the Cityscapes dataset with synthetic images that we generated with a more recent version of CARLA, creating our custom-made CCM dataset. As a result, we achieved a better accuracy of the semantic segmentation that the accuracy that the same models achieved after training only on real-world images of Cityscapes. We consider these results promising, as they indicate that improved semantic segmentation can be achieved with synthetic data by rather simple means, as we generated synthetic data for the CCM dataset by running simulations in available maps with the maximum number of assets (dynamic objects) possible on our data generation setup.

Furthermore, we also investigated how the amount of synthetic data used for augmentation affects the accuracy of the semantic segmentation. We did that by training DNN models on the three different splits of the CCM dataset containing all real-world images and 100, 50, and 25 percent of synthetic images, respectively. As a result, we found no direct correspondence between the amount of synthetic data and the quality of the semantic segmentation, as in the case of the MobileNetV2 model, the best result was achieved on the CCM-50 split, whereas in the case of Xception, the best result was achieved on the CCM-25 split. That indicates that attempting to improve the performance of a DNN model by simply augmenting the training dataset with a larger amount of synthetic data does not always work and suggests directions for future work on more elaborate methods of dataset augmentation with synthetic data.

## Figures and Tables

**Table 1:** Labelling for experiments with MICC-SRI dataset.

Cityscapes Labels	MICC-SRI Label	Labels for Augmented Dataset
Unlabelled, ego vehicle, rectification border, out of ROI, static, dynamic, rail track, sky, license plate	None, other	Other
Road	Road lines, roads	Roads
Ground, sidewalk, parking	Sidewalk	Sidewalk
Building	Buildings	Buildings
Wall, fence, guard rail, bridge, tunnel	Fences, walls	Fences, walls
Pole, pole group, traffic light, traffic sign	Poles, traffic signs	Poles, traffic signs
Vegetation, terrain	Vegetation	Vegetation
Person, rider	Pedestrian	Human
Car, truck, bus, caravan, trailer, train, motorcycle, bicycle	Vehicles	Vehicles

**Table 2:** Labelling for experiments with CCM dataset.

Cityscapes Label	CARLA (v0.9.12) Labels	CCM Dataset Labels
Unlabelled, ego vehicle, rectification border	Unlabelled	Unlabelled
building	Building	Building
Fence	Fence	Fence
Tunnel, pole group	Other	Other
Pedestrian, rider	Pedestrian, bike rider	Pedestrian and rider
Pole	Pole	Pole
Road	Road, roadline	Road
Sidewalk, parking	Sidewalk	Sidewalk and parking
Vegetation	Vegetation	Vegetation
Car, truck, bus, caravan, trailer, train, motorcycle, bicycle	Vehicles	Vehicles
Wall	Wall	Wall
Traffic sign	Traffic sign	Traffic sign
Sky	Sky	Sky
Ground	Ground	Ground
Bridge	Bridge	Bridge
Rail track	Rail track	Rail track
Guardrail	Guardrail	Guardrail
Traffic light	Traffic light	Traffic light
Static	Static	Static
Dynamic	Dynamic	Dynamic
Terrain	Water, terrain	Water and terrain

**Table 3:** Semantic segmentation (IoU) with MobileNetV2 on Cityscapes dataset and Cityscapes dataset augmented with MICC-SRI dataset.

Class	Cityscapes	Cityscapes Augmented with MICC-SRI
Road	**92.66**	92.62
Sidewalk	**67.02**	66.61
Building	**86.48**	86.18
Fences and Walls	**44.46**	43.21
Poles and traffic signs	**57.07**	56.72
Vegetation	**89.52**	89.45
Pedestrians	**76.59**	76.54
Vehicles	**89.65**	89.55
Mean IoU	**75.43**	75.11

**Table 4:** Semantic segmentation (IoU) with Xception on Cityscapes dataset and Cityscapes dataset augmented with MICC-SRI dataset.

Class	Cityscapes	Cityscapes Augmented with MICC-SRI
Road	**93.69**	93.60
Sidewalk	71.78	**72.70**
Building	**88.67**	88.30
Fences and Walls	**52.20**	49.16
Poles and traffic signs	**63.58**	62.52
Vegetation	**90.75**	90.58
Pedestrians	**81.75**	81.39
Vehicles	**92.29**	92.24
Mean IoU	**79.34**	78.81

**Table 5:** Semantic segmentation (IoU) with MobileNetV2 on Cityscapes and CCM datasets.

Class	Cityscapes	CCM-100	CCM-50	CCM-25
Building	75.54	79.39	**80.17**	79.18
Fence	00.02	21.49	**24.47**	17.82
Pedestrian and Rider	69.23	67.94	68.92	**69.38**
Pole	10.48	38.51	**38.54**	36.81
Road	88.57	88.46	**89.72**	89.15
Sidewalk	54.51	57.24	**59.79**	58.62
Vegetation	83.90	86.14	**86.41**	85.54
Vehicles	82.20	82.72	82.72	**82.78**
Wall	0.00	19.90	**23.81**	15.95
Traffic Sign	0.00	**35.42**	34.10	25.30
Sky	82.80	85.32	85.83	**85.85**
Traffic light	0.00	**21.32**	15.38	00.13
Water and Terrain	33.61	37.50	**38.73**	38.20
Mean IoU	44.68	55.49	**56.05**	52.67

**Table 6:** Semantic segmentation (IoU) with Xception on Cityscapes and CCM datasets.

Class	Cityscapes	CCM-100	CCM-50	CCM-25
Building	84.94	85.10	85.08	**85.62**
Fence	37.20	40.19	40.19	**43.44**
Pedestrian and Rider	**78.08**	76.42	76.94	77.92
Pole	45.08	48.50	48.75	**49.26**
Road	**92.31**	91.82	91.43	91.85
Sidewalk	65.80	67.21	67.09	**69.88**
Vegetation	87.71	87.00	87.59	**87.85**
Vehicles	**89.86**	88.82	89.63	89.63
Wall	23.29	28.88	27.69	**31.63**
Traffic Sign	44.42	50.89	55.83	**56.14**
Sky	85.13	88.79	89.70	**90.34**
Traffic Light	0.00	43.64	42.19	**44.13**
Water and Terrain	**46.86**	39.58	35.62	44.22
Mean IoU	57.25	63.14	63.87	**64.46**

## Data Availability

All datasets used in the present study as well as the Python scripts for preprocessing the data and training DNN models will be made available on request sent to the corresponding author’s email with appropriate justification.

## Abbreviations

The following abbreviations are used in this manuscript:

DNN	Deep neural network
GPU	Graphics processing unit
ROI	Region of interest
CNN	Convolutional neural network
MICC-SRI	Media Integration and Communication Center–Semantic Road Inpainting dataset
CCM	Cityscapes-CARLA Mixed dataset
IoU	Intersection over union
mIoU	Mean intersection over union
